# *Streptococcus suis* Induces Expression of Cyclooxygenase-2 in Porcine Lung Tissue

**DOI:** 10.3390/microorganisms9020366

**Published:** 2021-02-12

**Authors:** Muriel Dresen, Josephine Schenk, Yenehiwot Berhanu Weldearegay, Désirée Vötsch, Wolfgang Baumgärtner, Peter Valentin-Weigand, Andreas Nerlich

**Affiliations:** 1 Institute for Microbiology, Department of Infectious Diseases, University of Veterinary Medicine Hannover, Foundation, 30173 Hannover, Germany; muriel.dresen@tiho-hannover.de (M.D.); Josephine.Schenk@stud.mh-hannover.de (J.S.); Yeneb.Welde@tiho-hannover.de (Y.B.W.); desiree.voetsch@tiho-hannover.de (D.V.); 2 Institute for Pathology, University of Veterinary Medicine Hannover, Foundation, 30173 Hannover, Germany; Wolfgang.Baumgaertner@tiho-hannover.de; 3 Veterinary Centre for Resistance Research, Department of Veterinary Medicine, Freie Universität Berlin, 14163 Berlin, Germany

**Keywords:** *Streptococcus suis*, COX-2, cyclooxygenase, inflammation, lung infection, precision-cut lung slices

## Abstract

*Streptococcus suis* is a common pathogen colonising the respiratory tract of pigs. It can cause meningitis, sepsis and pneumonia leading to economic losses in the pig industry worldwide. Cyclooxygenase-2 (COX-2) and its metabolites play an important regulatory role in different biological processes like inflammation modulation and immune activation. In this report we analysed the induction of COX-2 and the production of its metabolite prostaglandin E_2_ (PGE_2_) in a porcine precision-cut lung slice (PCLS) model. Using Western blot analysis, we found a time-dependent induction of COX-2 in the infected tissue resulting in increased PGE_2_ levels. Immunohistological analysis revealed a strong COX-2 expression in the proximity of the bronchioles between the ciliated epithelial cells and the adjacent alveolar tissue. The morphology, location and vimentin staining suggested that these cells are subepithelial bronchial fibroblasts. Furthermore, we showed that COX-2 expression as well as PGE_2_ production was detected following infection with two prevalent *S. suis* serotypes and that the pore-forming toxin suilysin played an important role in this process. Therefore, this study provides new insights in the response of porcine lung cells to *S. suis* infections and serves as a basis for further studies to define the role of COX-2 and its metabolites in the inflammatory response in porcine lung tissue during infections with *S. suis*.

## 1. Introduction

*Streptococcus suis* is an important coloniser of the upper respiratory tract of pigs. It can cause a variety of disease manifestations ranging from pneumonia, septicaemia, meningitis to arthritis leading to great economic losses in the pig industry (reviewed in [1,2]). *S. suis* is also considered as a zoonotic agent that can cause meningitis and septicaemia [3,4]. The bacterium shows a high diversity of more than 700 sequence types and can be classified into different serotypes based on its capsule polysaccharide [5]. Although serotype 2 strains are considered the most common ones, causing infections in pigs and humans [1], infections with serotype 9 strains are becoming increasingly important in several countries, particularly in Western Europe [1,6]. Pathogenesis of *S. suis* infections is not completely understood, but a strong inflammatory response is a hallmark of *S. suis* infections. Some virulence and virulence-associated factors have been identified and characterised including the capsule, muraminidase-released protein, the extracellular factor and the pore-forming toxin suilysin (SLY) (reviewed in [7]). SLY belongs to the group of cholesterol-dependent toxins that are produced by numerous Gram-positive bacteria. Oligomerisation of the toxin subunits in cholesterol-rich membranes of host cells leads to the formation of large pores and consecutive cellular homeostatic changes culminating in immune activation and cell death [8].

Prostaglandin E_2_ (PGE_2_), a small molecular derivative of arachidonic acid produced by a variety of cell types, participates in different biological processes like inflammation modulation and immune activation [9]. In the lung, PGE_2_ is considered to play an important regulatory role in the control of inflammatory responses and tissue repair processes [10]. Generation of PGE_2_ starts with the liberation of arachidonic acid from cell membranes followed by conversion into prostaglandin H_2_ by the rate-limiting enzyme cyclooxygenase (COX), which can be further converted into PGE_2_ by PGE_2_ synthases [11,12]. Of the two isoforms, COX-1 is considered to be constitutively expressed and has homeostatic functions, whereas COX-2 is the inducible isoform involved in pathophysiological processes [11]. Released PGE_2_ can signal via four different receptors and elicit different immunomodulatory effects [13].

Induction of COX-2 and production of PGE_2_ were demonstrated in tissue biopsies from *S. pyogenes*-infected patients as well as in the tissue of experimentally infected mice [14] and were also found in human lung tissue infected with *S. pneumoniae* [15]. Furthermore, induction of COX-2/PGE_2_ was observed in swine infections with bacterial lung pathogens like *Mycoplasma hyopneumoniae* and *Actinobacillus pleuropneumoniae* [16,17]. Notably, to the best of our knowledge, COX-2 induction has not yet been reported for respiratory tract infections by *S. suis*. Thus, this study aimed at investigating whether COX-2, the rate-limiting enzyme of PGE_2_ production, is induced by *S. suis* in porcine lung tissue and at identifying bacterial factors that might contribute to induction.

## 2. Materials and Methods

### 2.1. Reagents

Precision-cut lung slice (PCLS) culture media were obtained from Thermo Fisher Scientific (Waltham, MA, USA) and Sigma-Aldrich (St. Louis, MO, USA). If not stated otherwise, all other reagents were from Sigma-Aldrich (St. Louis, MO, USA) or Roth (Karlsruhe, Germany). The experimental COX-2 inhibitor NS-398 and recombinant porcine interleukin 1β (IL1β) were purchased from Bio-Techne (Minneapolis, MN, USA). Fluorescently labelled phalloidin (Phalloidin-iFluor 647) was obtained from Abcam (Cambridge, UK). The following primary antibodies were used: monoclonal rabbit antibody against COX-2 (Clone SP21, Cat# SAB5500087-100UL, Sigma-Aldrich, St. Louis, MO, USA), mouse monoclonal anti-acetylated α-Tubulin antibody (Cat# sc-23950, RRID:AB_628409, St. Cruz (Dallas, TX, USA)), mouse monoclonal anti-vimentin antibody (Cat# GA630, RRID:AB_2827759, Agilent (St. Clara, CA, USA)) and rabbit monoclonal antibody against glyceraldehyde 3-phosphate dehydrogenase (GAPDH) (D16H11, RRID:AB_11129865, Cell Signaling Technology (Beverly, MA, USA)). Generation of the polyclonal antiserum against SLY was described previously [18]. Secondary goat anti-mouse immunoglobulin G (IgG) (H + L) Alexa Fluor^®^ 488 conjugated antibody (Cat# A-11029; RRID: AB_2534088), goat anti-rabbit IgG (H+L) Alexa Fluor^®^ 488 conjugated antibody (Cat# A-11008; RRID: AB_143165) and goat anti-rabbit IgG Alexa Fluor^®^ 568 (Cat# A-11011; RRID: AB_143157) were obtained from Thermo Fisher Scientific (Waltham, MA, USA). Secondary goat anti-rabbit IgG, horseradish peroxidase-linked (#7074, RRID:AB_2099233) was obtained from Cell Signaling Technology (Beverly, MA, USA).

### 2.2. Bacterial Strains and Recombinant Suilysin (SLY) Protein

The virulent, SLY-positive *S. suis* serotype 2 strain 10 (S10) was kindly provided by H. Smith (Lelystad, the Netherlands) [19]. Its isogenic SLY-deficient mutant (S10∆*sly*) was constructed by the insertion of an erythromycin cassette in the *sly* gene [18]. Complementation of S10∆*sly* (S10∆*sly*-C) was undertaken by allelic exchange and was described in a previous publication [20]. *S. suis* serotype 9 strain 8067 was isolated from a piglet and previously described [21]. All strains were grown on Columbia agar supplemented with 7% (*v*/*v*) sheep blood (Oxoid™, Thermo Fisher Scientific, Waltham, MA, USA) overnight at 37 °C under aerobic conditions. For infection experiments, cryo-conserved bacterial stocks were prepared from liquid cultures in Todd-Hewitt Broth (THB; Bacto™, Becton Dickinson, Franklin Lakes, NJ, USA) at the late-exponential growth phase (optical density at 600 nm = 1.1) as previously described [22]. Recombinant suilysin (rSLY) was expressed, purified and characterised as previously described [18].

### 2.3. Generation of Precision-Cut Lung Slice (PCLS) and Infection with Streptococcus suis (S. suis)

Precision-cut lung slices were prepared as previously described [22] from lungs of freshly slaughtered pigs (obtained from a local abattoir, Leine-Fleisch GmbH, Laatzen, Germany). In brief, the cranial, middle and intermediated lobes were carefully removed and the bronchi were filled with 37 °C low-melting agarose (GERBU, Heidelberg, Germany) in Roswell Park Memorial Institute (RPMI)-1640 medium (Sigma-Aldrich, St. Louis, MO, USA). After solidifying on ice, cylindrical portions of lung tissue were punched out with a tissue coring tool so that the bronchus/bronchiole was in the middle. The approximately 300-µm thick slices were cut by a Krumdieck tissue slicer (model MD 4000-01; TSE Systems, Chesterfield, MO, USA). PCLS were collected in RPMI-1640 medium (Thermo Fisher Scientific, Waltham, MA, USA) containing antibiotics and antimycotics (1 µg/mL clotrimazole, 10 µg/mL enrofloxacin (Bayer, Leverkusen, Germany), 50 µg/mL kanamycin, 100 U/mL penicillin, 100 µg/mL streptomycin/mL, 50 µg/mL gentamicin, 2.5 µg/mL amphotericin B). For removing the agarose, PCLS were bubbled with a normoxic gas mixture for two hours at 37 °C as previously described [23]. Then, the slices were transferred to 24-well plates (Greiner Bio-One, Kremsmünster, Austria) and incubated for one day in RPMI-1640 medium with antibiotics and antimycotics at 37 °C and 5% CO_2_. On the following day, viability of the slices was analysed by observing the ciliary activity using a DMi1 light microscope (Leica, Wetzlar, Germany). PCLS with ciliary activity of at least 80–90% were selected and washed twice with phosphate-buffered saline (PBS; Sigma-Aldrich, St. Louis, MO, USA). Afterwards, the slices were incubated in RPMI-1640 medium containing no antibiotics and antimycotics for another day.

**Figure 1:**
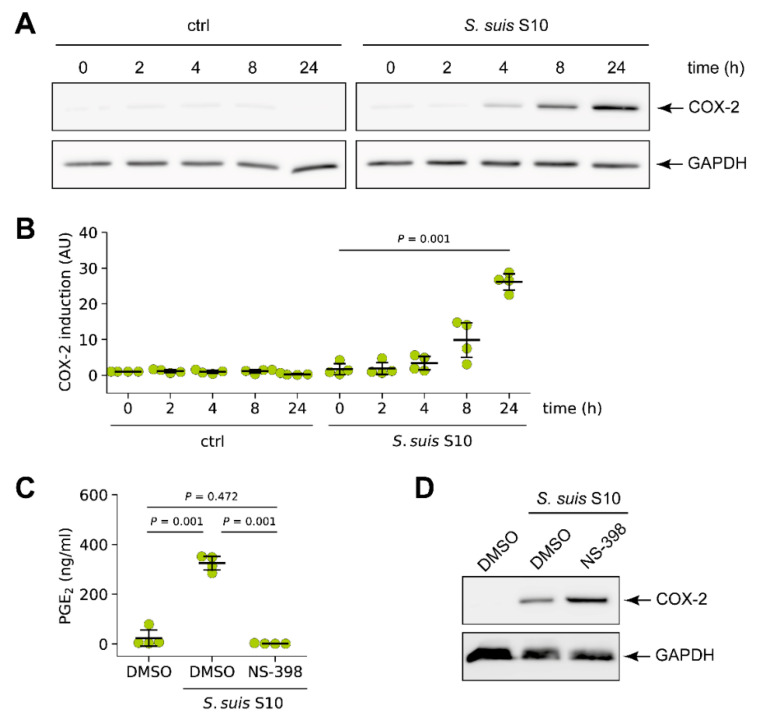
*S. suis* induced expression of cyclooxygenase-2 (COX-2) in porcine lung tissue. (**A**) Precision-cut lung slice (PCLS) were left uninfected (ctrl) or infected with *S. suis* S10 and COX-2 protein expression was analysed 0, 2, 4, 8, and 24 h after infection by Western blotting. Glyceraldehyde 3-phosphate dehydrogenase (GAPDH) served as loading control. One representative of four independent experiments is shown. (**B**) Densitometric analysis of the Western blot experiments described in (**A**). Data are presented as mean ± standard deviation (SD) of four independent experiments. Exact *p* values are indicated (Welch analysis of variance (ANOVA) followed by Games–Howell post hoc test). (**C**) PCLS were infected for 24 h with *S. suis* S10 in the presence of vehicle (DMSO) or the selective COX-2 inhibitor NS-398 (10 µM). Cells treated with vehicle (DMSO) alone served as control. Prostaglandin E_2_ (PGE_2_) production was determined in the supernatant by ELISA. Data are presented as mean ± SD of four independent experiments. Exact *p* values are indicated (Welch ANOVA followed by Games–Howell post hoc test). (**D**) Western blot analysis of COX-2 induction in PCLS treated and infected as described in (**C**). GAPDH served as loading control. One representative of three independent experiments is shown.

After washing the slices twice with PBS, they were inoculated with approximately 10^7^ CFU/well of *S. suis* S10, S10Δ*sly*, S10Δ*sly*-C and S8067 in 500 µL RPMI-1640, respectively. Slices were incubated for up to 24 h (h) at 37 °C and 5% CO_2_. In the experiments shown in Figure 1C,D, slices were left uninfected or infected for 24 h at 37 °C and 5% CO_2_ in the presence of 10 µM of the COX-2 inhibitor NS-398 or the vehicle dimethyl sulfoxide (DMSO). Supernatant and slices were collected at indicated time points for PGE_2_-enzyme-linked immunosorbent assay (ELISA), Western blot analysis and immunofluorescence staining. Slices were washed twice with PBS before collecting the samples. All experiments were performed in duplicate and repeated at least three times.

### 2.4. Isolatation of Primary Brochial Fibroblasts and Infection with S. suis

For isolating primary porcine bronchial fibroblasts, the bronchial tissue was carefully dissected, any parenchymal connective tissue was removed and the bronchus was cut into 5 mm thick rings. The pieces were placed in a 10 cm diameter cell culture dish in DMEM (Thermo Fisher Scientific, Waltham, MA, USA) supplemented with 10% fetal calf serum (FCS), 1% glutamine and antibiotics (100 U/mL penicillin, 100 µg/mL streptomycin, 50 µg/mL gentamicin, 2.5 µg/mL amphotericin B) and incubated at 37 °C and 5% CO_2_. After two weeks in culture, fibroblasts had grown out from the explanted tissue. The tissue pieces were removed and the cells were cultured for approximately two–three weeks until confluent cell islands were observed. Then, the cultures were passaged with a split ratio of 1:2 into 75 cm^2^ tissue culture flasks. Experiments were performed with cells between passages three and six showing typical fibroblast morphology and positive staining for vimentin by immunofluorescence microscopy. For infection with *S. suis*, fibroblasts were trypsinised, counted and 1.0 × 10^5^ cells per well were seeded on glass coverslips placed in a 24-well plate in Dulbecco’s modified Eagle medium (DMEM) with 10% FCS and 1% glutamine. After incubation at 37 °C and 5% CO_2_ over night, cells were infected for 8 h with *S. suis* using a multiplicity of infection (MOI) of one or five or left uninfected. As positive control, cells were stimulated with 40 ng/mL recombinant porcine IL1β for 8 h.

### 2.5. Sodium Dodecyl Sulfate Polyacrylamide Gel Electrophoresis (SDS-PAGE) and Western Blotting

For protein analysis by Western blotting, whole tissue lysates were prepared in cell extraction buffer (Thermo Fisher Scientific, Waltham, MA, USA) supplemented with protease inhibitor cocktail P8340, 1 mM 4-(2-aminoethyl)benzenesulfonyl fluoride hydrochloride and Halt phosphatase inhibitor cocktail (Thermo Fisher Scientific, Waltham, MA, USA) using an MP Biomedicals™ FastPrep-24™ 5G Instrument (St. Ana, CA, USA). Crude lysates were cleared by centrifugation and protein amounts were quantified by MicroBC Assay Protein Quantitation Kit (Interchim, Montluçon, France). Equal protein amounts (15 μg) were separated on 12% sodium dodecyl sulfate polyacrylamide gel electrophoresis (SDS-PAGE) gels which were then blotted onto polyvinylidene fluoride membranes. The latter were blocked in 5% (*w*/*v*) skimmed milk for 1 h at room temperature, washed and incubated overnight with the respective primary antibodies at 4 °C. After washing, the blots were incubated for 1 h with secondary antibodies against rabbit IgG and washed. All washing steps were performed in tris-buffered saline (TBS)/0.05% (*v*/*v*) Tween^®^ 20 (3 × 5 min). Blots were developed using SuperSignal West Pico Chemiluminescent Substrate (Thermo Fisher Scientific, Waltham, MA, USA) and a ChemoCam Imager 3.2 (Intas, Göttingen, Germany). Densitometry was performed using LabImage 1D version 4.1 (Kapelan Bio-Imaging, Leipzig, Germany).

### 2.6. Prostaglandin E_2_ (PGE_2_) Enzyme-Linked Immunosorbent Assay (ELISA)

PGE_2_ levels in the supernatants were determined using the PGE_2_ ELISA kit (Enzo Life Science, Farmingdale, NY, USA) in accordance with the manufacturer’s recommendations.

### 2.7. Immunofluorescence Staining of Histological Sections and Primary Fibroblasts

PCLS were fixed with 4% (*v*/*v*) formalin, embedded in paraffin blocks and sections of 3–4 µm were prepared. Sections were deparaffinised in Roti^®^Histol (Carl Roth, Karlsruhe, Germany), rehydrated in a descending series of ethanol (100%, 95%, and 70%) and cooked in sodium-citrate buffer (10 mM, pH 6.0, 10 min) for antigen retrieval. To block unspecific binding sides, the samples were incubated in 1% (*v*/*v*) bovine serum albumin (BSA), 5% (*v*/*v*) goat serum, 0.3% (*v*/*v*) Triton-X-100 and 0.05% (*v*/*v*) Tween^®^ 20 in PBS for 1 h at room temperature. All antibodies were diluted in 1% (*v*/*v*) BSA and 0.05% (*v*/*v*) Tween^®^ 20 in PBS and incubated for 1 h at room temperature or overnight at 4 °C. For visualing cilia, a monoclonal mouse antibody against acetylated α-tubulin (1:500), followed by an Alexa Fluor^®^ 488 goat-anti-mouse IgG (H + L) antibody (1:500), were used. COX-2 was stained using an anti-COX-2 antibody (1:100) and a goat-anti-rabbit Alexa Fluor^®^ 568 (1:500). Vimentin was visualised with a mouse monoclonal antibody diluted 1:100 in combination with an Alexa Fluor^®^ 488 goat-anti-mouse IgG (H + L) antibody (1:500). Cell nuclei were visualised by 4′, 6-diamidino-2-phenylindole (DAPI, 0.5 µg/mL in PBS, Cell Signaling Technology, Beverly, MA, USA). Stained sections were mounted with ProLong^®^ Gold Antifade Reagent (Cell Signaling Technology, Beverly, MA, USA) and stored at 4 °C until examination.

Fibroblasts were fixed with 3% (*v*/*v*) formaldehyde in PBS (methanol-free, Electron Microscopy Science, Hatfield, PA, USA) for 20 min at room temperature. Fixed cells were washed three times with PBS, permeabilised with 0.1% (*v*/*v*) Triton X-100 for 5 min and blocked in PBS containing 1% (*w*/*v*) BSA, 5% (*v*/*v*) goat serum and 0.05% (*v*/*v*) Tween^®^ for 30 min at room temperature. All antibodies were diluted in PBS containing 1% (*v*/*v*) BSA and 0.05% (*v*/*v*) Tween^®^ 20 and cells were washed for 5 min with PBS between staining steps. COX-2 was stained using an anti-COX-2 antibody (1:100, 1 h) and a secondary goat-anti-rabbit Alexa Fluor^®^ 488 antibody (1:500, 45 min) at ambient temperature. The actin cytoskeleton was stained with Phalloidin-iFluor 647 (1:500 in PBS) for 20 min at room temperature. Visualisation of nuclei and mounting was done as described above for PCLS.

### 2.8. Microscopy and Image Analysis

Confocal microscopy of PCLS was performed using a TCS SP5 confocal laser scanning microscope equipped with a 40 × 1.25-NA oil HCX Plan Apochromat objective and 63 × 1.40−0.60-NA oil HCX Plan Apochromat objective (Leica, Wetzlar, Germany). Overlapping image stacks with a z-distance of 1.0 µm per plane were acquired using a 1-Airy-unit pinhole diameter in sequential imaging mode to avoid bleed through. Maximum intensity projections were generated and the resulting images were stitched together using the pairwise stitching plugin [24] in Fiji-ImageJ version 1.53c [25]. Widefield microscopy of fibroblasts was performed with a Nikon Eclipse Ti-S microscope equipped with a 20 × 0.5-NA CFI Plan Fluor objective and a Nikon DS-QiMC-U2 camera controlled by NIS-Elements BR version 4.51.01 (Nikon, Tokyo, Japan). For display purposes, images were identically adjusted for contrast and brightness using Fiji-ImageJ version 1.53c.

COX-2 expression in fibroblasts was quantified using CellProfiler software version 4.0.7 (https://cellprofiler.org/, access date 23 January 2021) as previously described with modifications [26]. First, individual cells were identified by segmenting nuclei based on the DAPI signal. Next, the identified primary objects were propagated to obtain a mask for the cytoplasm. Subsequently, the intensity in the COX-2 channel was measured using these cytoplasm masks with the built-in measurement modules in CellProfiler. Measurements were exported and final data analysis was undertaken with Python 3.8 (Python Software Foundation, https://www.python.org/ (accessed on 30 September 2020)).

### 2.9. Statistical Analysis

Values are expressed as means ± SD from at least three independent experiments. Statistical analysis was performed with Python 3.8 (Python Software Foundation, https://www.python.org/ (accessed on 30 September 2020)) and the Pingouin (0.3.8) package [27]. Welch ANOVA followed by Games–Howell post hoc test was used for comparison of three or more groups.

## 3. Results

### 3.1. S. suis Induced Expression of Cyclooxygenase-2 (COX-2) in Porcine Lung Tissue

Studies using different streptococcal pathogens indicated an upregulation of COX-2 in streptococcal infections [14,15]. Since virtually no study has systematically analysed the biology of COX-2 in the porcine lung following *S. suis* infection, we used an ex vivo model of porcine precision-cut lung slices infected with *S. suis* to explore COX-2 induction and PGE_2_ synthesis.

To investigate whether COX-2 was expressed in *S. suis*-infected PCLS, slices were infected for the indicated time and COX-2 induction was analysed by Western blotting. In uninfected control slices, no induction of COX-2 expression was detectable during the course of the experiment. In contrast, in slices infected with *S. suis* S10 increasing amounts of COX-2 were detected over time, which started to rise at 4 h and continuously increased until the end of the experiment (Figure 1A). Densitometric analysis revealed a robust and significant induction after 24 h (Figure 1B).

Next, we determined PGE_2_ levels in *S. suis* infected PCLS after 24 h to test whether the increased expression of COX-2 translated into elevated COX-2 activity. The selective experimental inhibitor NS-398, that inhibits COX-2 activity and, thereby, production of prostaglandins by COX-2, was included to determine the amount of PGE_2_ produced by COX-2. As shown in Figure 1C, PGE_2_ was significantly increased in the supernatants of infected tissue compared with uninfected PCLS. This increase of PGE_2_ was completely blocked by treatment of PCLS with 10 µM NS-398, indicating that the synthesis of PGE_2_ in response to *S. suis* infection was mainly mediated by COX-2 (Figure 1C). Western blot analysis revealed an increase of COX-2 protein induction in the presence of the inhibitor compared to PCLS infected in the presence of solely DMSO (Figure 1D).

Taken together, the data indicate that COX-2 is strongly upregulated in porcine PCLS upon infection with *S. suis* resulting in the production of PGE_2_.

### 3.2. Localisation of COX-2 Induction in S. suis-Infected PCLS

In an ex vivo *S. pneumoniae* human lung infection model, alveolar type II cells, the vascular endothelium, and alveolar macrophages were identified as primary COX-2 producing cells [15]. To analyse which cells express COX-2 in our porcine PCLS model after infection with *S. suis*, we localised the COX-2 protein by immunohistochemistry.

**Figure 2:**
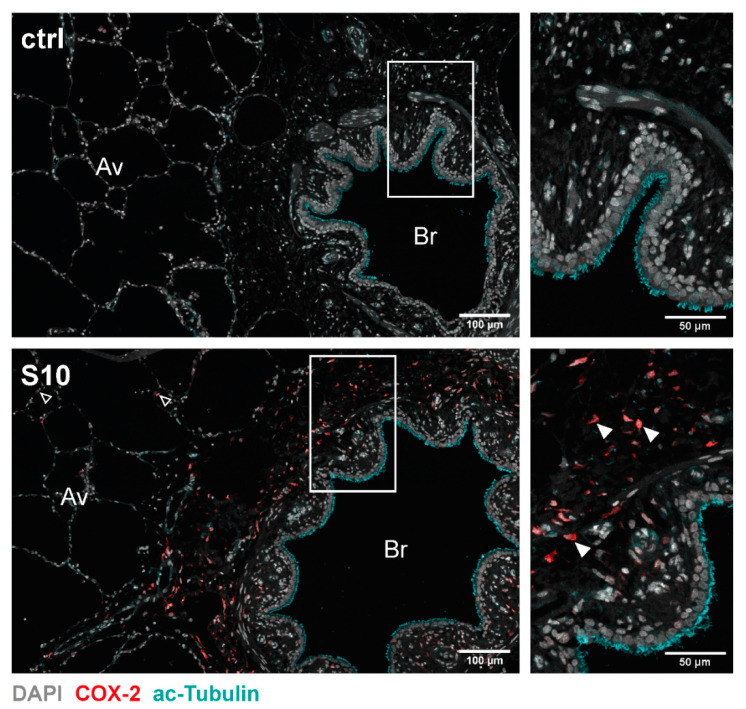
Localisation of COX-2 induction in *S. suis* infected PCLS. PCLS were left uninfected (ctrl) or infected with *S. suis* S10 (S10) for 24 h. After fixation the tissue was stained for COX-2 (red) and acetylated tubulin (cyan). Nuclei were visualised with DAPI (4′, 6-diamidino-2-phenylindole, grey). Stained tissue was analysed by confocal microscopy. Boxed regions are shown enlarged in the right panel. Open arrowheads indicate COX-2 expressing cells in the proximal alveolar tissue and closed arrowheads indicate exemplary COX-2 expressing cells in the bronchiolar region. One representative of three independent experiments is shown. Av, alveolar tissue; Br, bronchioles.

**Figure 3:**
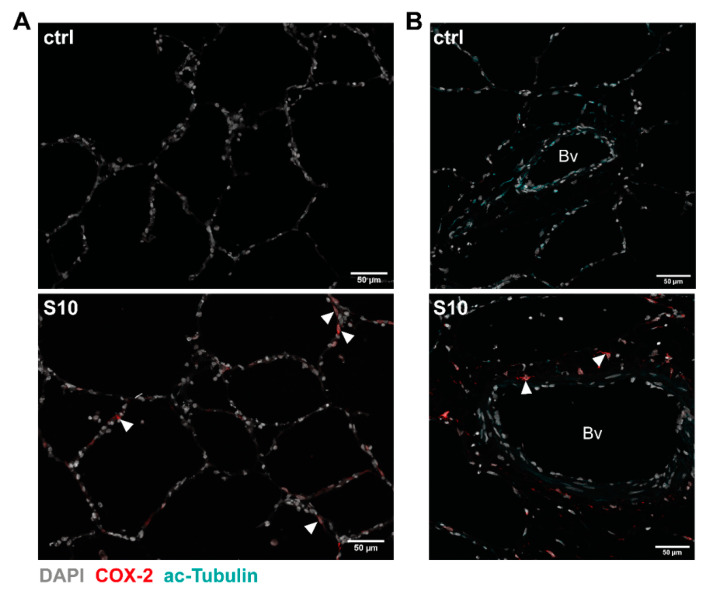
COX-2 induction in distal alveolar tissue and blood vessels in *S. suis* infected PCLS. PCLS were left uninfected (ctrl) or infected with *S. suis* S10 (S10) for 24 h. After fixation, the tissue was stained for COX-2 (red) and acetylated tubulin (cyan). Nuclei were visualised with DAPI (grey). Stained tissue was analysed for COX-2 induction in (**A**) distal alveolar tissue and (**B**) blood vessels by confocal microscopy. Arrowheads indicate exemplary COX-2 expression in the distal alveolar tissue ((**A**), S10) and in cells located in the basement membrane of the vessel ((**B**), S10). One representative of three independent experiments is shown. Bv, blood vessel.

**Figure 4:**
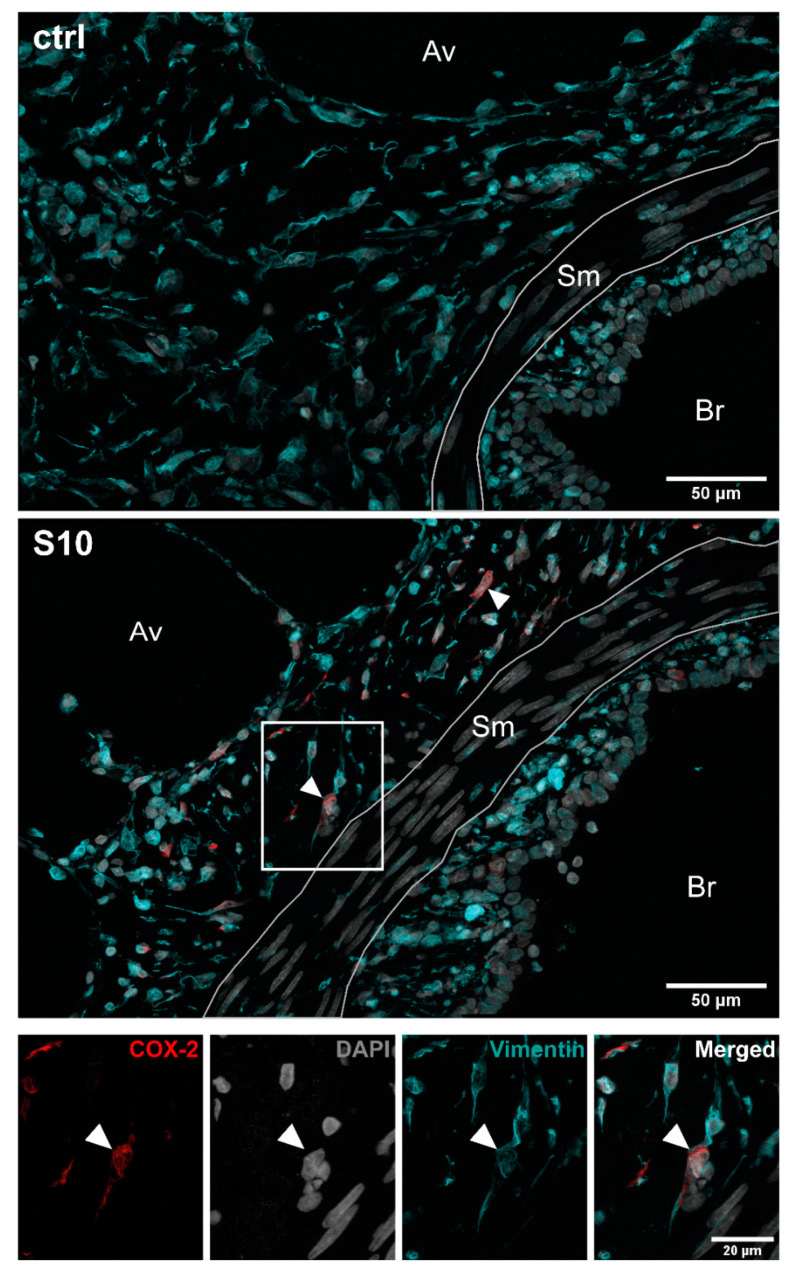
Co-staining of COX-2 and vimentin in *S. suis* infected PCLS. PCLS were left uninfected (ctrl) or infected with *S. suis* S10 (S10) for 24 h. After fixation, the tissue was stained for COX-2 (red) and vimentin (cyan). Nuclei were visualised with DAPI (grey). Stained tissue was analysed by confocal microscopy. The boxed region is shown enlarged in the lower panel. The area containing smooth muscle cells (Sm), which are negative for vimentin, is labelled. Closed arrowheads indicate exemplary elongated/spindle shaped cells in the bronchiolar region that show strong vimentin staining and are positive for COX-2. One representative of two independent experiments is shown. Av, alveolar tissue; Br, bronchioles; Sm, smooth muscle cells.

COX-2 staining was more abundant and intense in infected lung tissue than in uninfected control tissue (Figure 2). Close examination of the histological sections demonstrated that COX-2 was mainly expressed in cells located in a region between the ciliated cells of the bronchioles and the corresponding proximal alveolar tissue, representing the connective tissue surrounding the bronchioles (Figure 2, S10, closed arrowheads). We only found a low expression of COX-2 in a few cells in the adjacent alveolar tissue (Figure 2, S10, open arrowheads) as well as in the more distant alveolar tissue (Figure 3A, S10, closed arrowheads). Furthermore, examination of COX-2 induction in blood vessels did not show any induction in endothelial cells lining the lumen of the vessels, but we observed induction in some cells located in the basement membrane (Figure 3B, closed arrowheads). Finally, staining for vimentin, a widely used marker for fibroblasts, showed a strong signal in elongated/spindle-shaped cells positive for COX-2 in the bronchiolar region (Figure 4, S10, closed arrowheads).

To test whether COX-2 can be directly induced in fibroblasts by *S. suis*, we isolated primary porcine bronchial fibroblasts and infected them with *S. suis* S10 using two different multiplicities of infection. Stimulation with recombinant IL1β was used as positive control. As shown in Figure S1, we could not detect COX-2 induction by *S. suis* in isolated fibroblasts infected in vitro, while stimulation with IL1β resulted in a robust induction of COX-2 expression.

Taken together, vimentin staining and cell morphology/localisation suggest that after infection of PCLS with *S. suis*, COX-2 is most likely primarily produced in fibroblasts in the bronchiolar area. Lack of COX-2 induction in infected isolated fibroblasts hints towards indirect induction.

### 3.3. COX-2 is Induced by Two Prevalent S. suis Serotypes in Porcine PCLS

**Figure 5:**
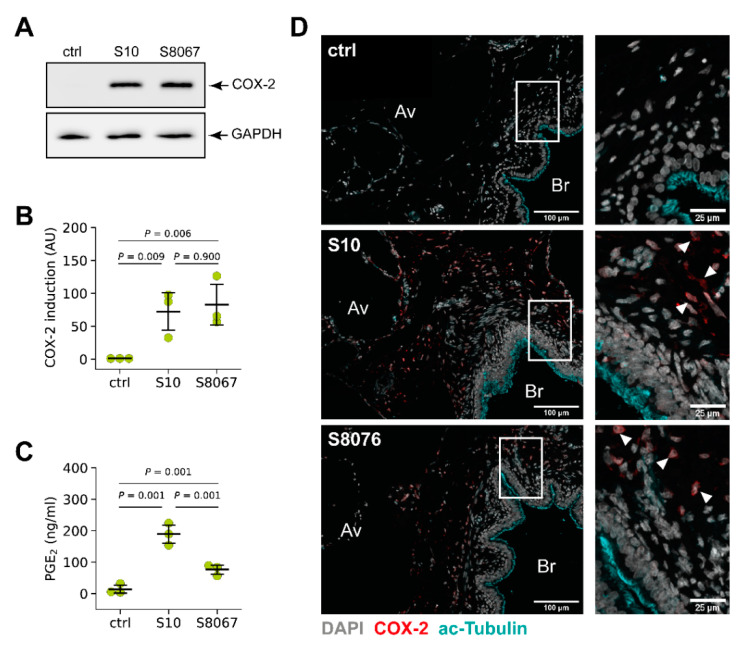
COX-2 is induced by two prevalent *S. suis* serotypes in porcine PCLS. PCLS were left uninfected (ctrl) or infected with *S. suis* S10 and S8067, respectively. (**A**) COX-2 protein expression was analysed 24 h after infection by Western blotting. GAPDH served as loading control. One representative of three independent experiments is shown. (**B**) Densitometric analysis of the Western blot experiments shown in (**A**). Data are presented as mean ± SD of three independent experiments. Exact *p* values are indicated (Welch analysis of variance (ANOVA) followed by Games–Howell post hoc test). (**C**) PGE_2_ production after 24 h of stimulation was determined in the supernatant by enzyme-linked immunosorbent assay (ELISA). Data are presented as mean ± SD of three independent experiments. Exact *p* values are indicated (Welch ANOVA followed by Games-Howell post hoc test). (**D**) Visualisation of COX-2 (red), acetylated tubulin (cyan) and nuclei (grey) in uninfected (ctrl) and infected (S10, S8067) tissue by confocal microscopy. Boxed regions are shown enlarged in the right panel. Arrowheads indicate exemplary COX-2 expressing cells. One representative of three independent experiments is shown. Av, alveolar tissue; Br, bronchioles.

To analyse whether COX-2 induction is specific for the serotype 2 strain S10, we next infected PCLS with the serotype 9 strain *S. suis* S8067 and compared COX-2 induction with that of *S. suis* S10. As shown in Figure 5A, Western blot analysis of infected PCLS after 24 h demonstrated a strong COX-2 induction in tissue infected with S8067 at a similar level as induced by S10, while no COX-2 induction was detectable in uninfected tissue. Densitometric analysis revealed a significant induction after infection with both strains when compared to control tissue, but no significant difference between the two strains (Figure 5B).

In line with increased COX-2 protein expression in PCLS infected with both strains, we also detected a significantly, 13.5-fold increased PGE_2_ level in tissue infected with S10 (Figure 5C). In tissue infected with S8076 we detected a 5.5-fold increased PGE_2_ level which is lower compared to infection with S10, but significantly increased compared to unstimulated PCLS (Figure 5C).

Immunohistochemical analysis of COX-2 induction in PCLS infected for 24 h revealed a similar localisation primarily in the bronchiolar region for both strains. Virtually no COX-2 induction was detectable in uninfected tissue (Figure 5D).

These results demonstrate that induction of COX-2 and PGE_2_ production in the PCLS model is not specific for *S. suis* S10, even though the level of produced PGE_2_ seems to vary between the two tested strains.

### 3.4. S. suis-Induced Expression of COX-2 in Porcine Lung Tissue is Modulated by SLY

Finally, we analysed the role of the pore-forming toxin SLY in the induction of COX-2 as a role for pore-forming toxins in this process has been demonstrated for *S. pyogenes* [28].

**Figure 6:**
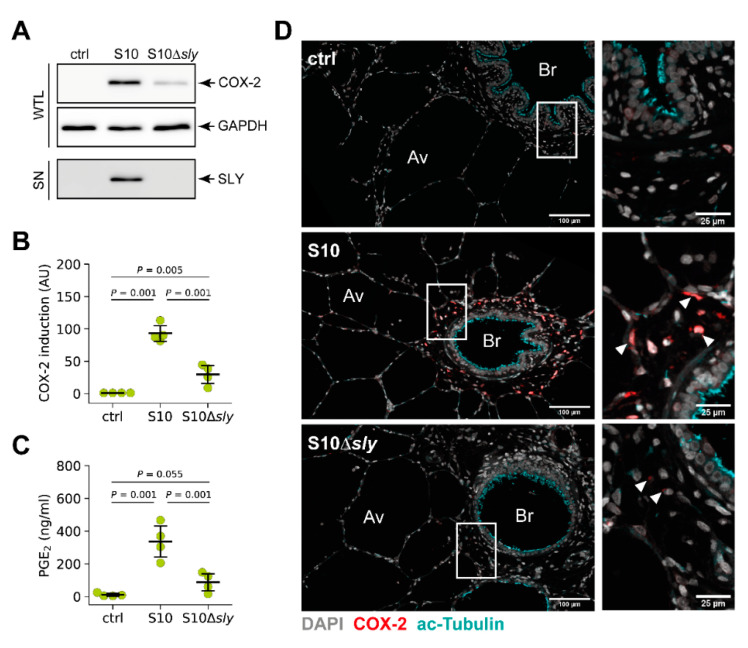
*S. suis*-induced expression of COX-2 in porcine lung tissue is suilysin (SLY)-dependent. PCLS were left uninfected (ctrl) or infected with *S. suis* S10 wild type and an S10Δ*sly* mutant, respectively. (**A**) COX-2 protein expression was analysed 24 h after infection by Western blotting of whole tissue lysates (WTC). GAPDH served as loading control. Expression of SLY was analysed by Western blotting of tissue culture supernatants (SN). One representative of four independent experiments is shown. (**B**) Densitometric analysis of COX-2 induction in the Western blot experiments shown in (A). Data are presented as mean ± SD of four independent experiments. Exact *p* values are indicated (Welch ANOVA followed by Games–Howell post hoc test). (**C**) PGE_2_ production after 24 h of stimulation was determined in the supernatant by ELISA. Data are presented as mean ± SD of four independent experiments. Exact *p* values are indicated (Welch ANOVA followed by Games–Howell post hoc test). (**D**) Visualisation of COX-2 (red), acetylated tubulin (cyan) and nuclei (grey) in uninfected (ctrl) and infected (S10, S10Δ*sly*) tissue by confocal microscopy. Boxed regions are shown enlarged in the right panel. Arrowheads indicate exemplary COX-2 expressing cells. One representative of three independent experiments is shown. Av, alveolar tissue; Br, bronchioles.

For this, PCLS were infected for 24 h with *S. suis* S10, its isogenic suilysin deletion mutant S10Δ*sly* or left uninfected. COX-2 induction was analysed by Western blotting of whole tissue lysates (WTL). As shown in Figure 6A, infection with the wild-type strain S10 induced COX-2 expression as demonstrated before. In contrast, tissue infected with S10Δ*sly* showed a reduced COX-2 protein level compared to tissue infected with the wild-type strain (Figure 6A, WTL). Densitometric analysis revealed a significant induction after infection with the S10 wild-type strain when compared to control tissue. Although we also observed a significant induction in case of S10Δ*sly* when compared to control tissue, there was significant difference in COX-2 induction between wild type and mutant by densitometry (Figure 6B). Furthermore, we proved production of SLY by the wild-type strain S10 and absence of SLY production by S10Δ*sly* by Western blotting of tissue culture supernatants (Figure 6A, SN). Reduced induction of COX-2 by S10Δ*sly* was restored by infection with a complemented suilysin mutant strain (Figure S2).

In accordance with reduced COX-2 protein expression, we observed a significantly reduced level of PGE_2_ in S10Δ*sly* infected PCLS compared to tissue infected with the wild-type strain S10 (Figure 6C).

Furthermore, immunohistochemical analysis of COX-2 induction in PCLS revealed a reduced number of COX-2 positive cells with a strongly reduced COX-2 signal in the tissue infected with S10Δ*sly* (Figure 6D).

**Figure 7:**
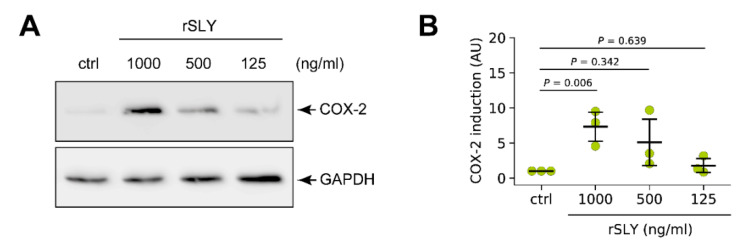
SLY is sufficient for COX-2 induction in porcine lung tissue. PCLS were left unstimulated or stimulated with 1000, 500 and 125 ng/mL recombinant suilysin (rSLY), respectively. (**A**) COX-2 protein expression was analysed 24 h after stimulation by Western blotting. GAPDH served as loading control. One representative of three independent experiments is shown. (**B**) Densitometric analysis of COX-2 induction in the Western blot experiments shown in (**A**). Data are presented as mean ± SD of three independent experiments. Exact *p* values are indicated (Welch ANOVA followed by Games–Howell post hoc test).

Finally, we tested whether suilysin alone is sufficient to induce COX-2 expression by stimulation of PCLS with increasing amounts of recombinant suilysin (rSLY) for 24 h and subsequent analysis of COX-2 induction by Western blotting. As shown in Figure 7A, stimulation with rSLY resulted in a dose-dependent increase of COX-2 expression. Densitometric analysis revealed a significant COX-2 induction in tissues stimulated with 1000 ng/mL rSLY (Figure 7B).

Taken together, these data indicate that the pore-forming toxin of *S. suis* plays an important role in induction of COX-2 expression and PGE_2_ production in PCLS.

## 4. Discussion

COX-2 generates metabolites that are important regulators of inflammation [9]. Studies using different streptococcal pathogens [14,15] indicated a prominent immunomodulatory role of COX-2 in streptococcal infections of murine and human skin as well as human lung tissue. Since virtually no study has systematically analysed the biology of COX-2 in the porcine lung following *S. suis* infection, we used an ex vivo model of porcine precision-cut lung slices infected with *S. suis* to explore COX-2 induction and PGE_2_ synthesis.

PCLS represent a model system with the typical lung architecture containing all relevant cell types maintained in their differentiation state and preserving important functions like ciliary activity of the bronchiolar epithelial cells [22]. This model also includes resident immune cells like dendritic cells and alveolar macrophages [29]. Therefore, this model is suitable to investigate the inflammatory response of the resident cells induced by interactions of the bacteria within the bronchial and alveolar compartment. However, not all aspects of immunity like recruitment of immune cells and resolution of the inflammatory response can be studied because the tissue is disconnected from the blood stream. Nevertheless, we observed a reproducible induction of COX-2 expression and production of PGE_2_ after infection with *S. suis* in this model that is comparable to results obtained with *S. pneumoniae* in a human lung ex vivo infection model [15] and might contribute to chemo- and cytokine regulation in the lung [9]. Interestingly and in contrast to the human lung ex vivo infection model, we observed an increase in COX-2 protein in the presence of the COX-2 inhibitor NS-398. This inhibitor mitigates the COX-2-mediated production of prostaglandins but does not inhibit the induction of COX-2. In one study using mouse lung fibroblast, PGE_2_ was shown to regulate COX-2 expression in a positive feedback loop [30], whereas other studies demonstrated a repressive effect of PGE_2_ in different cell/tissue systems [31,32]. The increase of COX-2 at the protein level in the presence of the inhibitor which almost completely reduces production of PGE_2_ in our model, also suggests a negative feedback loop.

We detected a strong COX-2 expression in the proximity of the bronchioles between the ciliated epithelial cells and the adjacent alveolar tissue. Morphology, location as well as the strong vimentin signal suggest that these cells are subepithelial bronchial fibroblasts. However, in future studies, staining with other fibroblast markers is required to further prove that these cells are indeed fibroblasts. We tested an antibody that is specific for human fibroblasts (TE-7 antibody) [33], but did not observe a specific staining in porcine lung tissue and isolated primary porcine fibroblasts, suggesting that the epitope recognised by the antibody is not present in porcine fibroblasts (data not shown). Lung fibroblasts play a key role in maintaining normal lung homeostasis and act as immune sentinels responding to inhaled toxic substances and cytokines like IL-1β [34]. Furthermore, a recent study demonstrated that fibroblast activity integrates innate immune signals to regulate the adaptive immune environment of the lung after infection with influenza A virus [35]. This study showed, that in virus-infected mouse lungs, three different classes of fibroblast can be distinguished: resting, ECM-synthesizing and inflammatory fibroblasts. Although COX-2 expression in the different classes of fibroblasts was not analysed in this study, it is tempting to speculate that the presence of functionally distinct fibroblasts may be the reason why we only detected COX-2 expression in a subset of vimentin-positive cells. However, to proof that these fibroblast subgroups are also present in our model system and that COX-2 induction correlates with these subgroups, single cell transcriptomic analysis needs to be performed in future studies.

Induction of COX-2 in fibroblasts is in contrast to studies on COX-2 induction in lungs of pigs infected with *M. hyopneumoniae* where induction was predominantly found in ciliated epithelial cells [16]. This might be explained by the direct interaction of *M. hyopneumoniae* with the ciliated bronchial cells and a lack of cytolytic toxins that primarily results in the activation of signalling pathways, subsequently leading to COX-2 induction in these cells [36]. Using a human lung ex vivo infection model, Szymanski et al. demonstrated that *S. pneumoniae* induces upregulation of COX-2 mainly in alveolar type II cells, but also in alveolar macrophages and endothelial cells [15]. We also detected COX-2-positive cells in the alveolar compartment, but the observed level of the COX-2 signal, as well as the number of positive cells, was much lower compared to the bronchiolar region. The reason for this difference could be the lack of bronchial structures in this human lung model system leading to an induction of COX-2 primarily in the alveolar tissue [15].

At the protein level, both strains induced strong expression of COX-2 while the PGE_2_ levels were lower in case of infection with the serotype 9 strain 8067. The final step of PGE_2_ generation initiated by COX-2 depends on the membrane-bound enzyme PGE_2_ synthase (mPGES) which can be induced by pro-inflammatory factors [12,37]. Although COX-2 and mPGES are thought to be functionally linked, the kinetics of COX-2 and mPGES induction are different in various cell types, suggesting different regulatory mechanisms controlling the expression of both enzymes [38,39,40]. While COX-2 expression can be directly induced by pore-forming toxins via, e.g., calcium signalling [28], induction of mPGES might be delayed due to lower induction of pro-inflammatory mediators required for its upregulation in case of infection with the serotype 9 strain 8067 and, therefore, leading to lower PGE_2_ levels. Interestingly, a recent study showed lower levels of pro-inflammatory cytokines in dendritic cells infected in vitro with a serotype 9 strain compared to infection with a serotype 2 strain [41]. However, due to the fact, that only two different strains were tested in the present study, more strains need to be analysed in follow-up studies to determine definitively whether there are strain-specific differences in COX-2 induction or PGE_2_ production.

COX-2 induction has been demonstrated for several different bacterial toxins, e.g., LPS [42], *C. difficile* toxin A [43] as well as the *S. pyogenes* cytolysins SLS and SLO [28]. SLY also belongs to the family of pore-forming toxins and exerts cytotoxic effects on various cell types such as epithelial cells, endothelial cells, phagocytes [18,44,45], but also on more complex tissue models like air–liquid interface cultures and PCLS [20,22]. In contrast to *S. pyogenes* expressing SLS and SLO, infection with a SLY-expressing *S. suis* strain does not induce COX-2 expression in isolated primary bronchial fibroblasts. However, in the study by Blaschke et al. [28] macrophages were used that, as professional phagocytic cells, might have the capacity to directly upregulate COX-2 after infection. Interestingly, we did not observe COX-2 expression in isolated fibroblasts after infection with *S. suis*, but after stimulation with recombinant IL1β. *S. suis* has been shown to induce IL1β in a SLY-dependent manner [46]. Therefore, it is tempting to speculate, that COX-2 induction is facilitated indirectly in the PCLS model via inflammatory mediators like IL1β and/or other cytokines produced by epithelial cells or resident immune cells in response to *S. suis* infection. Nevertheless, in this study, we provide evidence that SLY is involved in the induction of COX-2 and the synthesis of PGE_2_ in porcine lung tissue infected with *S. suis*, thus extending the list of functional activities of this toxin. In future, the regulatory mechanisms underlying the induction of COX-2 by *S. suis* should be further investigated.

## 5. Conclusions

In summary, this study demonstrated that *S. suis* induces COX-2 expression and PGE_2_ production in porcine PCLS. COX-2 induction is most likely primarily induced in fibroblasts located in the bronchiolar region. Furthermore, COX-2 is induced by two different *S. suis* strains representing prevalent serotypes and the cytolysin SLY is a critical factor involved in the induction of COX-2 and the synthesis of PGE_2_ in the porcine PCLS model. Further studies are needed to verify the obtained results in animal models and to analyse the role of COX-2 and PGE_2_ in the inflammatory response in the porcine lung during infections with *S. suis*.

## Supplementary Materials

The following are available online at https://www.mdpi.com/2076-2607/9/2/366/s1, Figure S1: COX-2 induction in primary porcine bronchial fibroblasts. Figure S2: Restoration of COX-2 expression in PCLS infected with the complemented suilysin mutant.
