# Supplementary material for: Streptococcus suis Induces Expression of Cyclooxygenase-2 in Porcine Lung Tissue
Source: Microorganisms. 2021 Feb 12;9(2):366. doi: 10.3390/microorganisms9020366 (PMC7917613; doi:10.3390/microorganisms9020366)
Supplement: Supplementary file 1 [file microorganisms-09-00366-s001.zip › Supplementary_Data/Figure_S1.pdf]

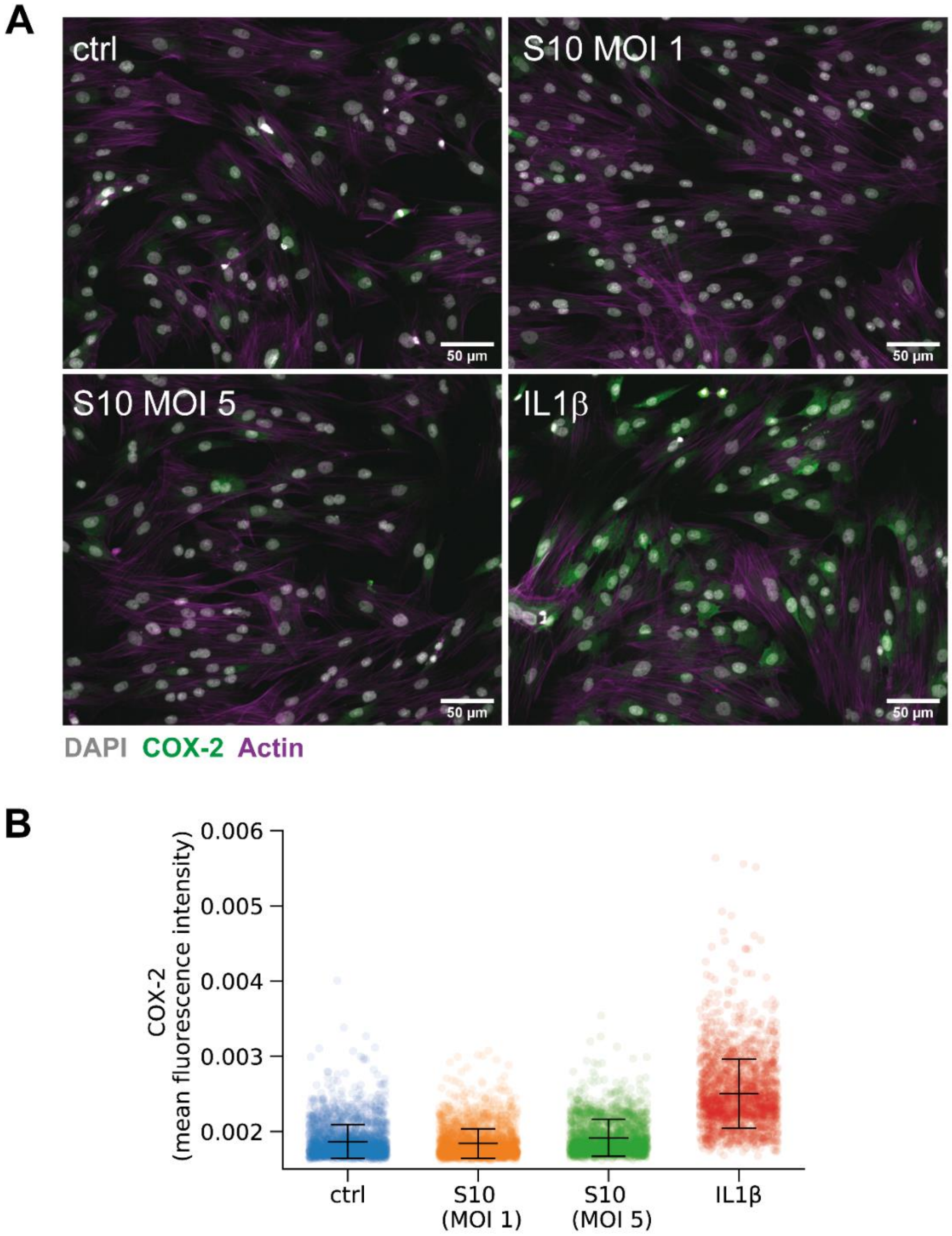

Figure S1. COX-2 induction in primary porcine bronchial fibroblasts. Primary bronchial fibroblasts were left uninfected (ctrl), infected with *S. suis* S10 wildtype at a multiplicity of infection of one (S10 MOI 1) or five (S10 MOI 5), respectively or stimulated with 40 ng/ml recombinant porcine IL1β for 8 h. **(A)** COX-2 (green) protein expression was analysed by immunofluorescence staining and widefield fluorescence microscopy. Nuclei were stained with DAPI (grey) and the actin cytoskeleton was visualised with fluorescently-labelled phalloidin (magenta). Representative images from one of two independent experiments are shown. **(B)** Quantification of COX-2 expression of cells stimulated/infected as described in (A). Cells in eight to ten randomly chosen positions were imaged and quantified per experiment. Pooled data from two independent experiments are displayed. In total 2144 (ctrl), 2475 (S10 MOI 1), 2209 (S10 MOI 5) and 1963 (IL1β) cells were analysed.
