# Supplementary material for: Streptococcus suis Induces Expression of Cyclooxygenase-2 in Porcine Lung Tissue
Source: Microorganisms. 2021 Feb 12;9(2):366. doi: 10.3390/microorganisms9020366 (PMC7917613; doi:10.3390/microorganisms9020366)
Supplement: Supplementary file 1 [file microorganisms-09-00366-s001.zip › Supplementary_Data/Figure_S2.pdf]

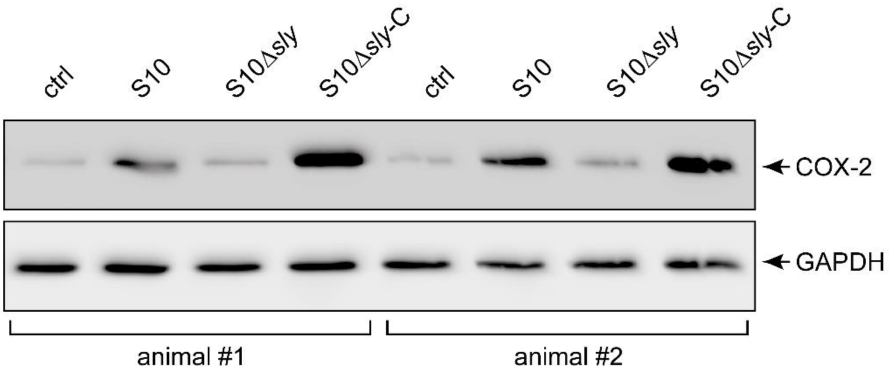

Figure S2. Restoration of COX-2 expression in PCLS infected with the complemented suilysin mutant. PCLS were left uninfected (ctrl) or infected with *S. suis* S10 wildtype (S10), a S10Δ*sly* mutant (S10Δ*sly*) and the complemented S10Δ*sly* mutant (S10Δ*sly*-C), respectively. COX-2 protein expression was analysed 24 h after infection by Western blotting of whole tissue lysates. GAPDH served as loading control. COX-2 induction in the tissue isolated from two different animals is shown.
